# Disseminated cutaneous *Mycobacterium avium* complex infection in a patient with GATA2 deficiency mimicking sarcoidosis

**DOI:** 10.1016/j.jdcr.2026.09.040

**Published:** 2026-09-29

**Authors:** Layan Shahrour, Jayson R. Miedema, Rachel Blasiak

**Affiliations:** a University of North Carolina School of Medicine, Chapel Hill, North Carolina; b Department of Dermatology, University of North Carolina, Chapel Hill, North Carolina

**Keywords:** cutaneous infection, GATA2 deficiency, granulomatous dermatitis, immunodeficiency, *Mycobacterium avium* complex, sarcoidosis mimic

## Introduction

Disseminated nontuberculous mycobacterial (NTM) infections are increasingly recognized in patients with underlying immunodeficiency. GATA2 deficiency, caused by germline GATA2 mutations, results in impaired monocyte, dendritic cell, and natural killer cell populations.^1^, ^2^ Clinically, patients may present with myelodysplastic syndrome, recurrent infections, lymphedema, and sensorineural hearing loss.^1^, ^2^ Disrupted interferon-γ-mediated immunity in GATA2 deficiency, which is critical for intracellular pathogen control, predisposes patients to opportunistic infections.^1^, ^2^, ^3^ Cutaneous involvement in NTM infection can present with nonspecific findings such as nodules or plaques and may mimic inflammatory or granulomatous conditions, leading to diagnostic delay. *Mycobacterium avium* complex (MAC) is the most frequently reported NTM in GATA2 deficiency and often presents with extrapulmonary or disseminated disease involving skin, lymph nodes, bone marrow, and lung.^1^, ^2^, ^3^ This reflects compromised monocyte-macrophage function and interferon-γ-dependent signaling required for effective mycobacterial control.^1^, ^2^ We present a case of disseminated cutaneous MAC infection in a patient with GATA2 deficiency initially suspected to have sarcoidosis, highlighting the diagnostic challenges posed by granulomatous skin lesions in immunocompromised patients.

## Case report

A 30-year-old woman with GATA2 deficiency-associated myelodysplastic changes, hearing loss, and immunosuppression had a prior diagnosis of pulmonary sarcoidosis treated with methotrexate and systemic corticosteroids. She presented with a 5-month history of warm, tender, erythematous nodules on the right arm and bilateral legs that were refractory to trimethoprim-sulfamethoxazole. She denied systemic symptoms beyond local tenderness, and she reported persistent lesion progression despite antimicrobial therapy.

Physical examination revealed red, indurated plaques with central firm papules on the proximal aspect of the right arm and bilateral legs (Fig 1, *A*). No mucosal involvement, fluctuance, drainage, or lymphadenopathy was identified. A skin biopsy of the right thigh was performed for histopathology (Fig 1, *B*).

**Fig 1:**
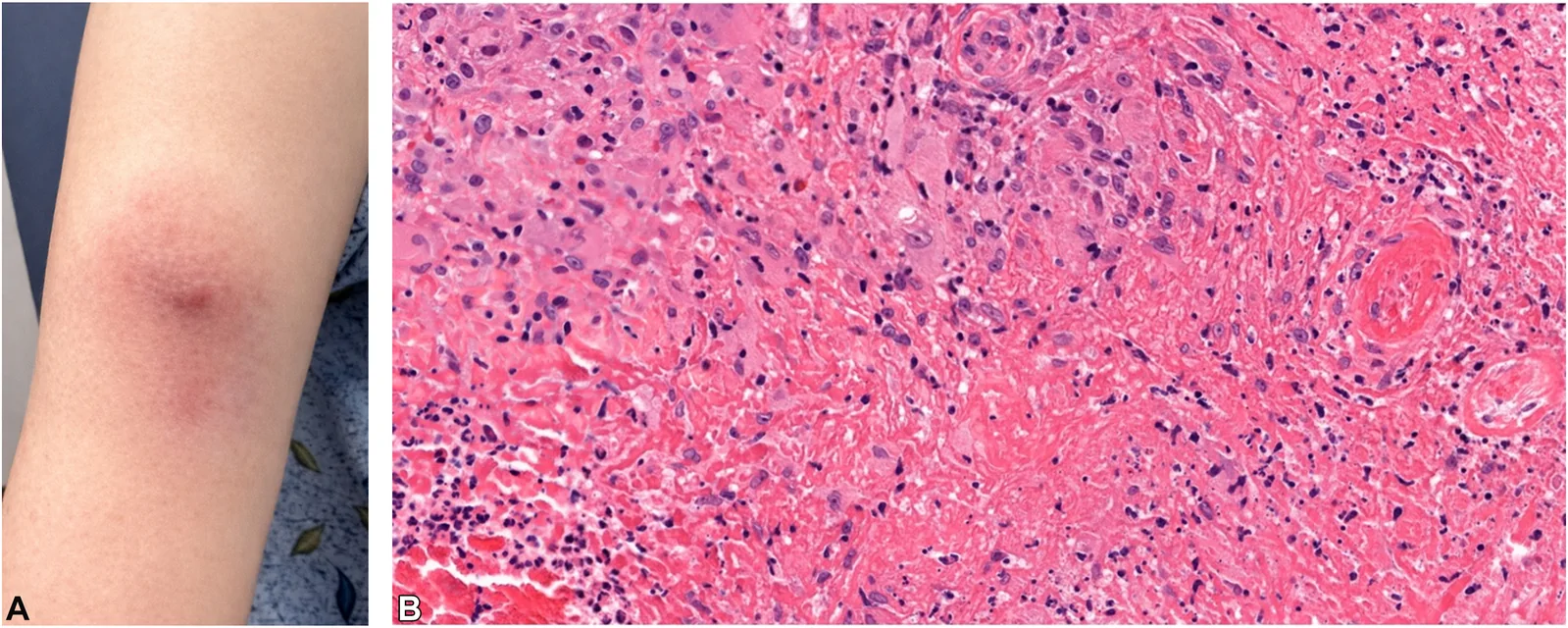
**A,** Clinical image of the proximal aspect of the right arm. **B,** Hematoxylin and eosin stain. **A,** Red, indurated plaques with central shiny papules on the right proximal arm. **B,** Results of the skin biopsy show deep dermal and subcutaneous necrotizing granulomatous inflammation with suppurative features. A mixed inflammatory infiltrate surrounds foci of necrosis and granuloma formation (original magnification: ×200).

Histopathology demonstrated deep dermal and subcutaneous suppurative and necrotizing granulomatous inflammation, and suspicious Fite-positive rod-like structures were identified on the Fite stain (Fig 2). Acid-fast bacilli smear was positive, and tissue cultures grew 3+ MAC, whereas aerobic and fungal cultures were negative. These findings and the patient’s cellular immunodeficiency make disseminated cutaneous MAC infection the most likely diagnosis. In immunocompromised hosts, organisms may be sparse on histologic stains, making culture confirmation essential for diagnosis.^4^, ^5^ Based on these findings, immunosuppressive therapy was discontinued and combination antimycobacterial therapy was initiated, and the patient subsequently underwent allogeneic stem cell transplantation.

**Fig 2:**
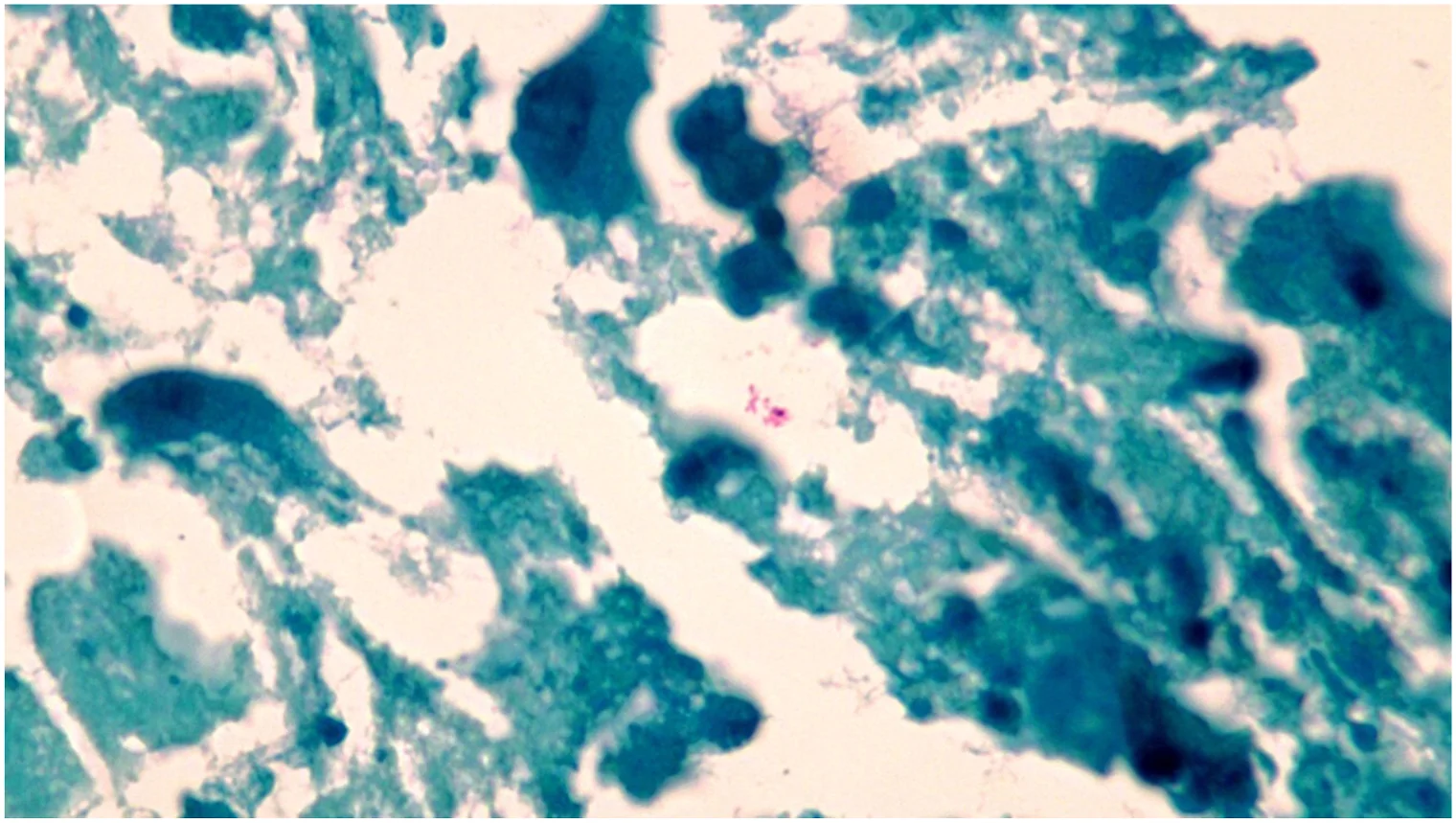
Fite stain of skin biopsy showing disseminated cutaneous *Mycobacterium avium* complex infection. Fite stain of skin biopsy highlighted small red-staining acid-fast bacilli within areas of necrotizing granulomatous inflammation. Although organisms are few and not definitive morphologically, subsequent tissue culture confirmed *M*. *avium* complex (original magnification: ×1000).

## Discussion

This case highlights the importance of considering disseminated NTM infection in immunocompromised patients with persistent granulomatous skin lesions, particularly when the clinical presentation resembles inflammatory dermatoses. These findings and the patient’s cellular immunodeficiency make disseminated cutaneous MAC infection the most likely diagnosis.

The principal diagnostic challenge in this case was distinguishing disseminated MAC infection from other causes of granulomatous or panniculitic skin lesions. The differential diagnosis for disseminated cutaneous MAC infection includes cutaneous sarcoidosis, erythema nodosum, and subcutaneous panniculitis-like T-cell lymphoma. Cutaneous sarcoidosis classically demonstrates nonnecrotizing granulomas with minimal surrounding inflammation and no detectable organisms. Microbiologic cultures are negative, and immunosuppression typically improves lesions.^6^ In contrast, this patient demonstrated necrotizing granulomatous inflammation, positive acid-fast staining, and MAC growth on tissue culture, which strongly favors disseminated MAC infection over sarcoidosis. Reports also describe patients initially diagnosed with sarcoidosis who were later found to have NTM infection, particularly when disease is refractory to corticosteroids or occurs in immunodeficient hosts.^6^, ^7^

Erythema nodosum is clinically characterized by tender erythematous nodules, most often on the shins, but histologically represents septal panniculitis without vasculitis, granulomatous necrosis, or detectable microorganisms.^8^ The presence of necrotizing granulomas and positive mycobacterial cultures makes erythema nodosum substantially less likely.

Subcutaneous panniculitis-like T-cell lymphoma presents with nodules and plaques and shows lobular panniculitis with atypical cytotoxic lymphocytes rimming adipocytes, often accompanied by systemic symptoms.^9^ Infectious organisms are absent, and cultures would be negative.^9^ The absence of atypical lymphocytes and positive cultures excludes this diagnosis.

Management of disseminated MAC infection may require prolonged combination therapy with a macrolide plus ethambutol, with or without a rifamycin.^5^ When feasible, immunosuppressive medications, including corticosteroids and methotrexate, should be discontinued because they may increase the risk of disseminated mycobacterial infection.^5^ For patients with GATA2 deficiency, allogeneic hematopoietic stem cell transplantation remains the only curative therapy for the underlying immune defect and is recommended in those with myelodysplastic syndrome or recurrent severe infections.^1^, ^10^ Recognition of this uncommon presentation may facilitate earlier diagnosis and appropriate management in similarly immunocompromised patients.

In conclusion, this case demonstrates disseminated cutaneous MAC infection presenting as necrotizing granulomatous nodules in a patient with GATA2 deficiency, initially mimicking sarcoidosis. Because organisms may be sparse on histologic stains, negative or equivocal staining should not preclude obtaining mycobacterial cultures when clinical suspicion remains high. This case highlights the importance of maintaining a broad differential diagnosis in immunocompromised patients with granulomatous skin lesions. Appropriate reevaluation as needed and early involvement of a multidisciplinary team are some pivotal factors in determining successful treatment outcomes.

## Conflicts of interest

None disclosed.
